# Initial pediatric robot‐assisted adrenalectomy using the hinotori Surgical Robot System

**DOI:** 10.1111/ped.70271

**Published:** 2025-12-08

**Authors:** Yuichi Okata, Jun Teishima, Tadashi Hatakeyama, Masao Yasufuku, Kandai Nozu, Hideaki Miyake, Yuko Bitoh

**Affiliations:** ^1^ Division of Pediatric Surgery, Department of Surgery Kobe University Graduate School of Medicine Kobe Japan; ^2^ Division of Urology, Department of Surgery Related Kobe University Graduate School of Medicine Kobe Japan; ^3^ Department of Pediatric Surgery Hyogo Prefectural Kobe Children's Hospital Kobe Japan; ^4^ Department of Pediatric Surgery Kakogawa Central City Hospital Kakogawa Japan; ^5^ Department of Pediatrics Kobe University Graduate School of Medicine Kobe Japan

**Keywords:** child, hinotori Surgical Robot System, pediatric adrenalectomy, pheochromocytoma, robot‐assisted surgery

Robot‐assisted surgery (RAS) has been increasingly applied in pediatric patients using systems, such as the da Vinci Surgical System.^1^ These systems offer precision and 3D visualization, but most are designed for adults and are difficult to apply in small children.^2^ This report presents the first successful case of pediatric adrenalectomy using the Japanese hinotori™ Surgical Robot System.^3^, ^4^ Beyond its novelty, this case demonstrates how novel robotic systems can be thoughtfully adapted to the pediatric population.

An 11‐year‐old child (145 cm, 34 kg), representing a typical school‐aged child, was diagnosed with a 40‐mm right adrenal pheochromocytoma. Preoperative imaging confirmed a well‐circumscribed tumor without vascular invasion or distant metastasis. Given the patient's small body size and the deep retroperitoneal location of the adrenal gland, a robotic approach was considered feasible but required meticulous planning. A patient‐specific port placement simulation was performed using 3D reconstruction based on CT data. This allowed visualization of spatial relationships between organs and predicted arm movements, ensuring safe and interference‐free docking. Preclinical validation with infant‐sized models showed that the compact articulated arms and open console design allowed safe maneuvering without collisions in narrow operative fields, advantageous in pediatric settings (Figure 1).^3^, ^4^, ^5^

**FIGURE 1:**
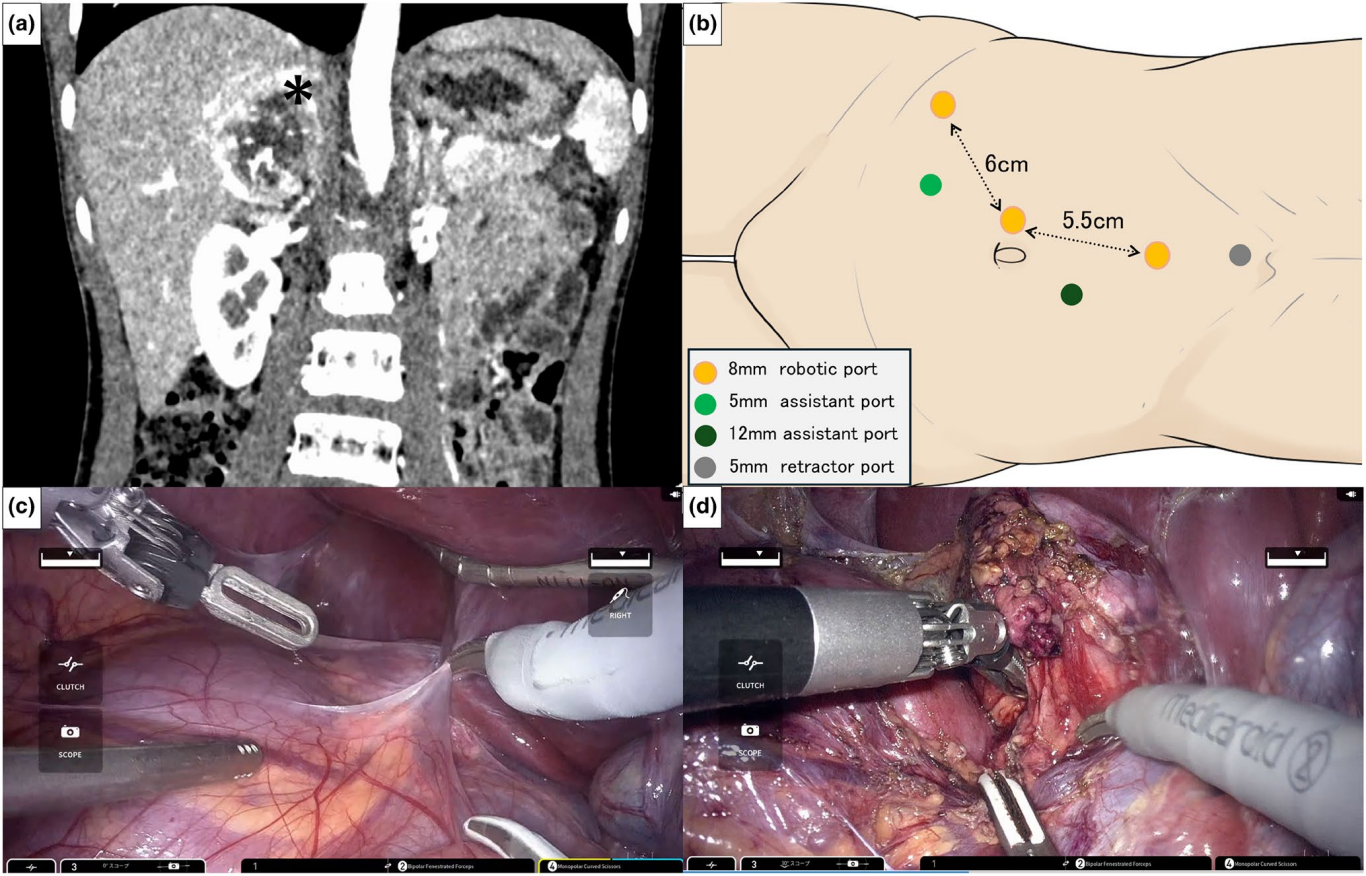
Preoperative imaging, port placement, and intraoperative views of robot‐assisted adrenalectomy using the hinotori™ Surgical Robot System. The procedure was performed on September 18, 2024. (a) Coronal contrast‐enhanced CT scan showing a right adrenal tumor (*). (b) Schematic diagram of port placement. Three 8‐mm robotic ports (orange), one 12‐mm assistant port (dark green), one 5‐mm assistant port (light green), and one 5‐mm retractor port (gray) were placed. (c) Initial intraoperative view showing port orientation and retroperitoneal dissection. (d) Tumor mobilization using robotic fenestrated bipolar forceps and monopolar curved scissors.

The procedure was performed under general anesthesia with the patient in the left lateral decubitus position. Based on the simulation, three 8‐mm robotic ports, one 12‐mm assistant port, one 5‐mm assistant port, and one 5‐mm retractor port were placed. After pneumoperitoneum was established, the robotic arms were docked. Monopolar scissors and bipolar forceps were used. The adrenal gland was carefully dissected under high‐definition 3D vision. The adrenal vein was clipped and divided securely, and the specimen was extracted through an extended incision at the 12‐mm assistant port, which was enlarged by approximately 1.5 cm to allow tumor removal.

The operative time was 2 h 57 min, with a console time of 1 h 24 min. Estimated blood loss was minimal, and no transfusion was required. Intraoperative hemodynamic fluctuations characteristic of pheochromocytoma were observed but successfully controlled. The flexible articulation of the robotic instruments allowed precise dissection with minimal manipulation of the tumor, which helped avoid excessive catecholamine release and contributed to stable intraoperative hemodynamics.

Although drainage was <10 mL/day on POD1, the drain was left longer due to tumor vascularity and was removed after hemodynamics stabilized.

Histopathological analysis confirmed complete tumor resection with negative margins and no signs of malignancy. Because histology alone cannot exclude malignancy in pheochromocytoma, we emphasized the need for long‐term surveillance. Genetic testing for pheochromocytoma‐related mutations was performed and revealed no pathogenic variants.

This case illustrates that robot‐assisted adrenalectomy using the hinotori™ Surgical Robot System is both technically feasible and clinically safe in a school‐aged child. Success was supported by patient‐specific simulation and flexible instruments, which enabled precise dissection without compressing the tumor, which is advantageous in pheochromocytoma.

While a direct comparison with the da Vinci system was not performed in this study, our focus was on demonstrating the feasibility and safe introduction of the hinotori™ Surgical Robot System in pediatric surgery. Comparative studies are warranted in the future.

Unlike widely used systems, hinotori™ Surgical Robot System was developed in Japan and allows close collaboration between surgical teams and domestic engineers. This enabled rapid feedback and iterative adjustments during the preclinical phase. The cost‐effectiveness of the system may also make it more suitable for pediatric use in regions where the number of pediatric surgical cases is limited, such as in Japan's declining birthrate environment.

This initial case demonstrates that careful preoperative simulation, multidisciplinary planning, and cautious perioperative management can enable safe adaptation of new robotic systems to pediatric surgery; however, as a single case, it does not imply universal applicability.

RAS in pediatric surgery requires careful patient selection, structured training, multidisciplinary collaboration, and adaptation of tools and techniques. Most current robotic platforms lack pediatric‐specific instruments, which limit their flexibility. Nonetheless, this experience highlights that thoughtful customization of adult‐designed systems can create new possibilities for pediatric surgical innovation.

As the first pediatric case with hinotori™ Surgical Robot System, this report highlights both technical success and the strategic steps needed for safe adaptation. Future work should include refinement of instruments, development of pediatric‐specific training, multicenter data collection, and integration of engineering support with simulation as standard practice. As robotic platforms diversify globally, sharing structured pathways for adaptation may support safer and more equitable access to RAS for children worldwide.

## AUTHOR CONTRIBUTIONS

Y.O. conceived and designed the study, served as the primary surgeon, collected and analyzed the data, and took the lead in writing and revising the manuscript. J.T. supervised the surgical procedure. Y.B. provided general supervision of the surgical strategy. T.H. advised on surgical indication and perioperative planning. M.Y. participated in patient management and contributed to surgical planning. K.N. and H.M. supervised the study from a clinical and academic perspective. All authors reviewed and approved the final manuscript.

## CONFLICT OF INTEREST STATEMENT

Jun Teishima belongs to an endowed chair funded by Medicaroid Corporation. All other authors declare no conflict of interest.

## INFORMED CONSENT

Informed consent was obtained from the patient's guardian for the publication of this case report, including the use of clinical data and intraoperative images.

## Data Availability

The data that support the findings of this study are available from the corresponding author upon reasonable request.

## References

[1] Pham HD , Okata Y , Vu HM , Tran NX , Nguyen QT , Nguyen LT . Robotic‐assisted surgery for choledochal cyst in children: early experience at Vietnam National Children's hospital. Pediatr Surg Int. 2019;35:1211–1216. 31270674 10.1007/s00383-019-04518-w

[2] LP O'B , Hannan E , Antao B , Peirce C . Paediatric robotic surgery: a narrative review. J Robot Surg. 2023;17:1171–1179. 36645643 10.1007/s11701-023-01523-z PMC10374698

[3] Hinata N , Yamaguchi R , Kusuhara Y , Kanayama H , Kohjimoto Y , Hara I , et al. Hinotori surgical robot system, a novel robot‐assisted surgical platform: preclinical and clinical evaluation. Int J Urol. 2022;29:1213–1220. 35851692 10.1111/iju.14973

[4] Motoyama D , Matsushita Y , Watanabe H , Tamura K , Otsuka A , Fujisawa M , et al. Robot‐assisted adrenalectomy using a hinotori surgical robot system: report of first series of six cases. Asian J Endosc Surg. 2023;16:489–495. 37231618 10.1111/ases.13212

[5] Kameoka Y , Okata Y , Yoshimura S , Inuzuka S , Iwabuchi S , Miyauchi H , et al. Evaluation of the hinotori(™) surgical robot system for accurate suturing in small cavities. J Robot Surg. 2024;18:294. 39068349 10.1007/s11701-024-02053-y PMC11283413

